# Highlighting the Importance of Characterization Techniques Employed in Adsorption Using Metal–Organic Frameworks for Water Treatment

**DOI:** 10.3390/polym14173613

**Published:** 2022-09-01

**Authors:** Thabiso C. Maponya, Katlego Makgopa, Thabang R Somo, Kwena D. Modibane

**Affiliations:** 1 Nanotechnology Research Lab, Department of Chemistry, School of Physical and Mineral Sciences, University of Limpopo (Turfloop), Sovenga 0727, Polokwane, South Africa; 2 Department of Chemistry, Faculty of Science, Tshwane University of Technology (Arcadia Campus), Pretoria 0001, South Africa

**Keywords:** adsorption, analytical techniques, heavy metals, metal–organic frameworks

## Abstract

The accumulation of toxic heavy metal ions continues to be a global concern due to their adverse effects on the health of human beings and animals. Adsorption technology has always been a preferred method for the removal of these pollutants from wastewater due to its cost-effectiveness and simplicity. Hence, the development of highly efficient adsorbents as a result of the advent of novel materials with interesting structural properties remains to be the ultimate objective to improve the adsorption efficiencies of this method. As such, advanced materials such as metal–organic frameworks (MOFs) that are highly porous crystalline materials have been explored as potential adsorbents for capturing metal ions. However, due to their diverse structures and tuneable surface functionalities, there is a need to find efficient characterization techniques to study their atomic arrangements for a better understanding of their adsorption capabilities on heavy metal ions. Moreover, the existence of various species of heavy metal ions and their ability to form complexes have triggered the need to qualitatively and quantitatively determine their concentrations in the environment. Hence, it is crucial to employ techniques that can provide insight into the structural arrangements in MOF composites as well as their possible interactions with heavy metal ions, to achieve high removal efficiency and adsorption capacities. Thus, this work provides an extensive review and discussion of various techniques such as X-ray diffraction, Brunauer–Emmett–Teller theory, scanning electron microscopy and transmission electron microscopy coupled with energy dispersive spectroscopy, and X-ray photoelectron spectroscopy employed for the characterization of MOF composites before and after their interaction with toxic metal ions. The review further looks into the analytical methods (i.e., inductively coupled plasma mass spectroscopy, ultraviolet-visible spectroscopy, and atomic absorption spectroscopy) used for the quantification of heavy metal ions present in wastewater treatment.

## 1. Introduction

Fresh water is one of the vital needs that is required for life on Earth. Unfortunately, due to the contamination of fresh water by several industrialized activities, water scarcity is a challenge across the globe [1,2]. Predictions have shown that there are possibilities that some areas will have serious water shortages by 2025 [3]. To overcome this challenge, the reusability of wastewater has become the ideal way to conserve and increase the availability of water in their reserves. The advantages of using recycled wastewater include irrigating agricultural soil, aquaculture, manufacturing consumption, recreational and environmental practices, as well as artificial groundwater recharge [1,2]. In general, recycled wastewater can be utilized in these processes and replace fresh water, provided a suitable purification procedure is implemented to acquire the desired water quality for the specified application [2]. Various water pollutants that are found in water bodies are classified according to their origin, with the main classifications being organic, biological, and inorganic [2,4,5]. Heavy metals (which form part of the inorganic pollutants) are the most investigated due to their persistence and non-biodegradable nature. Many of these types of contaminants are found to be toxic and carcinogenic, and can accumulate through food chains causing very serious health hazards to living organisms [6,7]. Furthermore, some heavy metals are of economic importance such as platinum group metals (PGMs), and therefore, their conservation and recycling have become important in order to meet future demands [8,9]. In an attempt to find solutions to these problems, scientists have investigated and developed several technologies including chemical precipitation, flocculation/coagulation, reverse osmosis, membrane filtration, and adsorption [6]. Due to the cost-effectiveness and simplicity of the adsorption method, in this review, we focus more on this method to narrate the instrumental techniques used to understand the structural effects of MOFs in wastewater treatment.

Adsorption is the process by which mass transfer occurs between substances at the interface of two phases. The phases in adsorption can either be liquid–solid, liquid–liquid, gas–liquid, or gas–solid. The solutes of gas or liquid (referred to as adsorbate) accumulate on the surface of an adsorbent (either solid or liquid) [10]. Depending on the properties and the constituents of the adsorbate and the adsorbent, there are two possibilities for the adsorption process that can take place. The first one is physisorption (adsorptive adsorption), which occurs when there is a physical adsorbate–adsorbent contact either through van der Waals, London, or dipole–dipole interactions [11,12]. These types of interactions are weak and can be reversed. Secondly, an interaction can occur between an adsorbent and an adsorbate which can result in the formation of a chemical bond referred to as chemisorption (reactive adsorption) [10,13,14]. Chemisorption takes place only on monolayers and the adsorbate–adsorbent interaction cannot be easily broken due to strong forces acting between them [10,11]. Factors that contribute to the type of adsorption process taking place are chemical structures such as functional groups and physical structures including specific surface area and pore size [15]. Generally, the adsorption process takes place naturally in many physical, biological, and chemical systems. Furthermore, this process is applicable in numerous industrial applications such as separation, purification of gases, [16,17], isolation of biological compounds [18], drug delivery [19] as well as in wastewater treatment [14,20].

The utilization of adsorption technology in the remediation of polluted water is considered to be a feasible way to recycle wastewater by removing pollutants. The adsorption technique has enjoyed widespread attention in wastewater treatment due to the following advantages: cost-effectiveness, ease of implementation and operation, high efficiency, and the possibility to regenerate the adsorbent material since it is reversible [3,6]. Moreover, it allows for the use of a wide range of adsorbent materials and generates less harmful secondary products, which can be used for other applications. The process operates in the liquid phase where the dissolved pollutants (adsorbates) are transferred from the liquid state (wastewater) to the surface of an adsorbent, which, in many cases, is a solid material. The result of this mass transfer produces clean water which can be reused for other suitable applications [3]. For wastewater treatment through adsorption, the parameters that are taken into consideration include the properties (chemical and physical) of the characteristics of the adsorbent and adsorbate, effects of temperature, interaction time, solution pH, amount of adsorbent, the initial concentration of adsorbate in liquid, as well as the effect of competing ions [21]. These parameters are the ones used when determining the adsorption capacities of adsorbent materials toward the targeted pollutant. In addition, the effectiveness of this method is deduced from the ability of the adsorbent to selectively target certain molecules, its reusability, and regeneration [3,15,22].

Metal–organic frameworks (MOFs) are currently in the spotlight of research due to their intrinsic properties that make them suitable for use in various applications. These materials are composed of metal ion centers that are connected to one another by organic linkers to achieve a variety of structural geometries [23,24]. Owing to the intriguing properties (i.e., high specific surface area, tunable pore size, high porosity, and crystalline structure), MOFs have been explored in various applications such as gas sensors, energy storage, drug delivery, and water purification [25,26,27]. Furthermore, their surface functionality allows the incorporation of other functional materials to form composites. Recently, MOFs have attained great attention as suitable adsorbents for the recognition and elimination of heavy metal ions in wastewater treatment [23,28]. The presence of heavy metal ions in wastewater poses serious health threats to living organisms, even at low concentrations. One of the major steps in treating water that is contaminated with heavy metal ions is to determine and measure the initial concentrations of the pollutants that are present in the water. After the adsorption process has taken place, the efficiency/adsorption capacity of the adsorbent (i.e., MOFs) is obtained by measuring the remaining concentrations of heavy metal ions after separating the adsorbent from the adsorbate aqueous solution. The heavy metals that have been widely studied using MOFs and their composites as adsorbents include lead (Pb), chromium (Cr), rare earth elements (REE), mercury (Hg), arsenic (As), copper (Cu), cobalt (Co), nickel (Ni), and platinum group metals (PGMs). Analytical methods can be employed to determine the concentrations of heavy metals before and after adsorption. In addition, the adsorption mechanism can also be revealed by characterization of the MOF adsorbent material before and after adsorption using spectroscopic, morphological and physical techniques. However, the poor solubility of MOFs in most organic solvents hinders the characterizations of MOF using some spectroscopic techniques. MOFs can easily be damaged by high energy electron irradiation using transmission electron microscopy. The most primitive MOFs have the inherent defects of poor electrical conductivity and low structural stability, which impact their practical performance.

In this review paper, we present some of the literature on the analytical techniques employed in studying and better understanding the adsorption behavior of MOFs toward the removal of heavy metal ions in wastewater. Initially, a brief background on water pollution by heavy metal ions as well as MOFs as possible adsorbents is introduced in relation to the adsorption technology in wastewater treatment. This is followed by a discussion and comparison of different techniques such as UV-Vis, ICP-MS, ICP-OES, and FAAS to analyze and to determine the remaining concentration of the heavy metals before and after adsorption. Furthermore, various methods of characterization that assist in deducing the structures of MOF composites/nanocomposites and their possible interactions with heavy metals in the adsorption process are discussed.

## 2. Analytical Methods for Heavy Metal Analysis

### 2.1. Inductively Coupled Plasma Mass Spectrometry

Inductively coupled plasma mass spectrometry (ICP-MS) is a powerful technique that combines both the advantages of ICP and a mass spectrometer to give elemental analysis data [29]. The incredible properties of this instrument include multiple elemental analysis ability, adequate precision, and lower detection limits. Furthermore, ICP-MS has a longer linear dynamic range, uncomplicated spectra, and the capability to rapidly analyze isotopes [29,30,31].

#### System Operation

The working principle of the ICP-MS is based on three main components:

##### Sample Introduction and Conversion to Ions

In this step, the analyte (in liquid form) is introduced into the system through suction by a peristaltic pump which provides a constant uptake flow rate. In this case, an autosampler is used to optimize the analysis time and to reduce the consumption of reagents. The sample is pumped into the nebulization chamber, where it gets converted from a liquid solution to aerosol. The smallest droplets are carried to the plasma by the help of argon gas, whereas the larger droplets are drained out of the system.

##### ICP Compartment

Initially, the analyte aerosol is filtered prior to being introduced into the plasma to prevent overloading the solvent which may result in plasma extinction. The argon plasma interacts with the electromagnetic field provided by the radiofrequency source and it becomes generated on top of a torch. Once the aerosol is injected into the plasma, it instantaneously dissolves, vaporizes, atomizes, and subsequently ionizes depending on the ionization potential of the individual elements. The argon plasma provides heat with high temperatures from 6000 to 10,000 K. Due to its chemical inertness and higher ionization energy that emit a simple spectrum, argon gas is widely used in ICP. It is capable of ionizing or exciting many elements of the periodic table without the formation of a stable compound with the analyte [32].

##### Mass Spectrometer and Detection System

**Figure 1:**
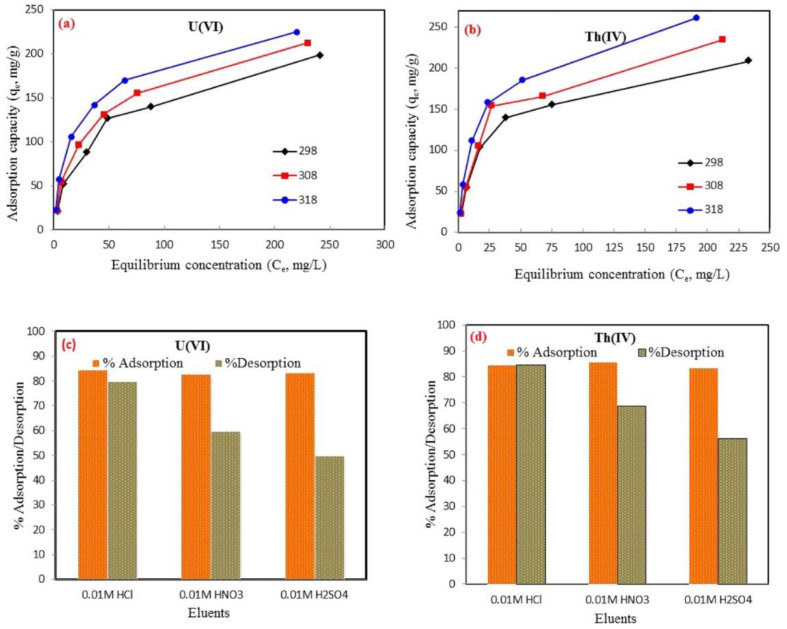
Temperature effect of the adsorption of (**a**) U(VI) and (**b**) Th(IV) ions by Fe_3_O_4_@AMCA-MIL53(Al) nanoadsorbent. Regeneration and reusability cycles towards (**c**) U(VI) and (**d**) Th(IV) metal ions [35].

The vaporized ions and atoms are then carried by argon gas from the ICP torch to the interface. The compartment of the interface is composed of two subsequent cones (skimmer and sampler) which permit the ions to focalize into a smaller volume. Followed by their introduction into the mass spectrometer, either a quadrupole or hexapole, they are separated based on their mass to charge ratio and then move to the detector. In ICP-MS, a detector is mostly an electron multiplier that works by converting ion signals to electric pulses [29,31,32,33,34]. The ICP-MS technique is adequate to quantitatively identify metal ions present in natural and drinking waters at trace levels, which are of particular relevance for toxicity control regions that may have been contaminated by toxic metals. ICP-MS has been recognized as a suitable method for adsorption/water treatment as it provides good sensitivity, requires minimal sample size, affords minimal elemental interferences, and readily provides a means to perform rapid and automated multi-elemental analyses. For example, an ICP-MS analytical instrument was employed in the work reported by Alqadami and colleagues [35]. The authors synthesized a nanocomposite of Fe_3_O_4_@AMCA-MIL53(Al) for the adsorption of thorium Th(IV) and uranium U(IV) ions from simulated wastewater. Adsorption experiments were carried out by mixing 0.02 g of Fe_3_O_4_@AMCA-MIL53(Al) with 25 mL of Th(IV) and U(VI) solutions containing 20 mg L^−1^ for 24 h. For determining the maximum adsorption capacity, the initial concentrations of Th(IV) and U(VI) ranged between 20 and 400 mg L^−1^ with the optimum solution pH of 4.7 and 5.5 for Th(IV) and U(VI), respectively. Different temperatures of 25, 35, and 45 °C were investigated at a contact time of 300 min. The remaining concentrations of the analyte after adsorption were obtained from the ICP-MS analysis and the data are represented in Figure 1. For both Th(IV) and U(VI), the Fe_3_O_4_@AMCA-MIL53(Al) adsorbent showed increasing values of equilibrium adsorption capacities (Q_e_) as the temperatures and initial concentrations increased (see Figure 1a,b).

Furthermore, the regeneration studies of the prepared Fe_3_O_4_@AMCA-MIL53(Al) were investigated by adsorption/desorption experiments and 25 mL of various acidic eluents of 0.01 M were used to regenerate the adsorbent material. The concentrations of Th(IV) and U(VI) were determined using ICP-MS and calculated from Equation (1):


(1) $$\%adsorption=\frac{Co-Ce}{Co}\;\times \;100$$

The results obtained are represented in Figure 1c,d. On the basis of the data, the authors were able to conclude that 0.01 M HCl provided more desorption efficiency for Th(IV) and U(VI) as compared with HNO_3_ and H_2_SO_4_ of the same concentration. The use of ICP-MS technique for adsorption studies provides the advantages of a smaller sample size, element-specific information, quantitation, rapid sample throughput, and significantly higher recovery of all elements of interest, especially the volatile elements. ICP-MS is characterized by the following advantages such as high sensitivity analysis, lower detection limits of most elements (ppt or ppq-range), simultaneous multi-element analysis; wide dynamic range, and isotope composition. The disadvantages of ICP-MS are the high operational costs because of the high amount of argon used and the high susceptibility to high salt concentrations present in digest solutions or in sweat and saliva extraction solutions, resulting in interferences of the measurements.

### 2.2. Inductively Coupled Plasma Optical Emission Spectroscopy

Inductively coupled plasma optical emission spectroscopy (ICP-OES) was developed in the 1960s and first commercialized in the mid-1970s [33]. The sample introduction process in ICP-OES is analogous to one of the ICP-MS instruments. However, in ICP-OES, once the plasma has ionized, the analyte atoms move to the excited state and emit light energy upon their return to the ground state [33]. This light energy, which is emitted by metal atoms/ions, is transformed into an electrical signal followed by detection and quantitative measurements from a photoelectron multiplier tube (PMT). ICP-OES has advantages for the detection of heavy metals in water; however, this technique is limited by the need to transform a solid sample into a solution which is time consuming as it requires over 60% of the total time to complete the analysis. Thus, considerable improvement is required in this regard as there is a weak link in heavy metal analysis to ensure that the analytes are completely released and solubilized, i.e., total decomposition of the sample is achieved.

**Figure 2:**
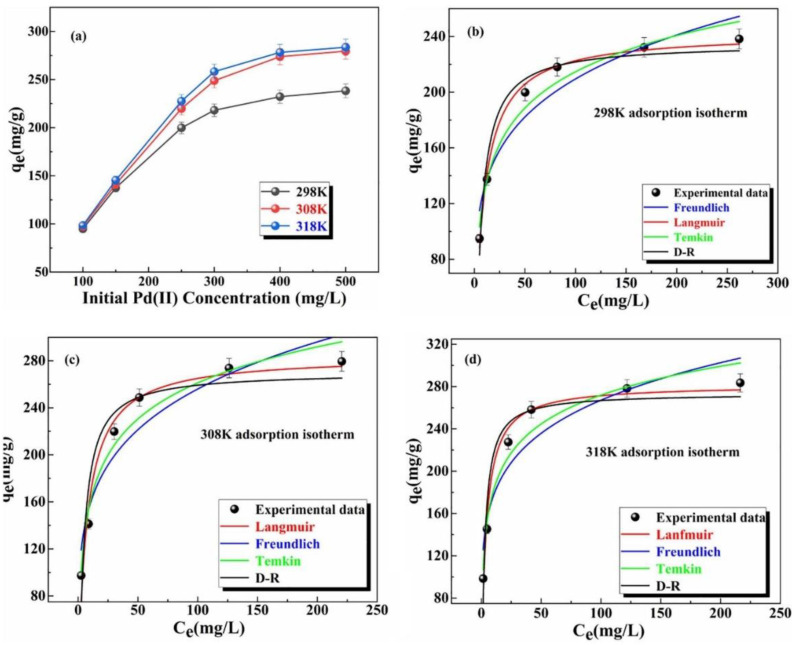
(**a**) Effect of Pd(II) initial concentration. Data fitting of adsorption isotherm models at: (**b**) 298 K; (**c**) 308 K; (**d**) 318 K [36].

The recent work reported by Tang et al. [36] showed that pre-modification of Zr-based MOF with 4-amino-3-hydroxybenzoic acid and p-phthalaldehyde (AHPP) was effective in removing Pd(II) pollutants in simulated wastewater. They conducted their batch adsorption experiments by contacting 0.01 g of AHPP-MOF with 10 mL of 100 mg L^−1^ Pd(II) solution at a pH of 4. The samples were centrifuged for 24 h at a speed of 280 rpm. The remaining concentrations of the Pd(II) ion were determined from the ICP-AES, Leeman Prodigy7, United States. The effects of initial concentration ranging from 100 to 600 mg L^−1^ and temperatures of 298, 308, and 318 K were used to determine the adsorption capacity. As shown in Figure 2a, their results showed that the adsorption capacity increased as both the temperature and original concentration increased.

Furthermore, the data obtained were fit to four different isotherm models (Langmuir, Freundlich, Temkin, and D-R), for the determination of maximum adsorption capacity (Q_max_), as shown in Figure 2b–d. The data fit the Langmuir more than others with Q_max_ values of 241.6, 288.48, and 293.65 mg g^−1^ at 298, 308, and 318 K, respectively. Therefore, the advantages of ICP-OES over an atomic absorption spectrometer and UV-VIS are that both simultaneous and sequential analyses of multiple elements is possible, the calibration function is spread over a wide dynamic range, and the number of measurable elements is high. However, one of the disadvantages of the ICP-OES method is the high argon consumption.

### 2.3. Flame Atomic Absorption Spectrometer

The spectroscopy of flame atomic absorption (FAAS) is one of the popular techniques that is utilized when determining the metal element concentrations present in particular analytes. The method was initially developed in 1952 and only became commercialized as an analytical technique in the 1960s [37] The FAAS technique was directly developed from atomic absorption spectroscopy (AAS) which is based on the theory of atoms/ions having the ability to absorb light at a particular wavelength that is unique [38].

The main principle in FAAS involves the atomization of a solution containing the analyte using a flame. Firstly, the analyte (in solution form) is introduced into the system through an inlet tube into the nebulizer where the liquid is converted into small droplets (aerosol mist), followed by introduction into the flame [39,40]. Then, the flame atomizes the sample elements to their ground state atoms that are free and prone to excitation. A hollow cathode lamp provides pure light with a specific wavelength and energy which passes through the flame, and is absorbed by atoms/ions of the element of interest. Upon absorption of the light energy, the electrons in the atoms become excited and jump to higher energy levels. The radiation leaves the sample cell and goes to the monochromator where it is separated into wavelengths that are detected by a PMT. This is followed by intensification and conversion of the photon signals to electrons, which is quantified as electric current [41,42]. In AAS, these measurements assist in calculating the amount of the element present in an analyte in terms of absorbance and/or concentration [43]. The relationship between light absorption and the concentration of the element is described by the Beer–Lambert law, which assumes direct proportionality between them under certain conditions. To determine the unknown concentration of an analyte, a calibration curve is required which is obtained from the standard of a known concentration and more than 62 metal element concentrations can be obtained [39,44,45].

**Figure 3:**
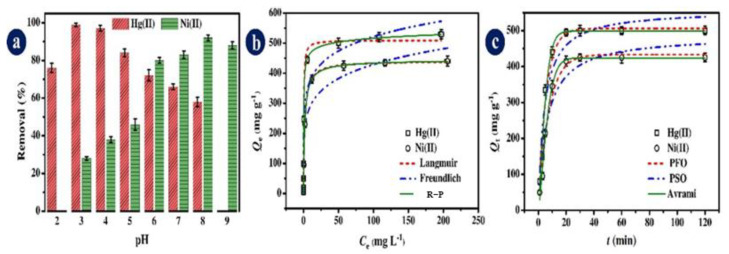
(**a**) Solution pH influence; (**b**) original Hg(II) and Ni(II) concentration with isotherm models fit; (**c**) interaction time with kinetic models fit [46].

FAAS is preferred for determining trace levels of metal ions in environmental samples due to its simplicity and cost-effectiveness as compared with other instrumental techniques, such as ICP-OES and ICP-MS. However, analytes at lower levels than the detection limit of AAS and large amounts of salt in the real samples are the two primary limitations in determining metal ion levels though AAS. Such techniques are not sufficiently sensitive and selective for certain analyses. Thus, methods for separating or preconcentrating trace elements may be necessary before spectrometric analysis. In the work reported by Soltani and colleagues [46], a nanocomposite of layer-double hydroxide LDH/MOF was synthesized and employed in the adsorption of divalent mercury, Hg(II), and nickel, Ni(II) ions. For the adsorption experiments, they used a constant amount of 2.0 mg of LDH/MOF nanocomposite, which was contacted with 20 mL of Hg(II) and Ni(II) at a temperature of 22 ± 3 °C and 200 rpm shaking speed. The main adsorption variables investigated were (see Figure 3): (a) solution pH, (b) primary metal ions concentration, and (c) the interaction time. After the adsorption process took place, the LDH/MOF nanocomposite was separated from the Hg(II) and Ni(II) ions solutions by centrifuging at a speed of 5000 rpm. Then, the filtrates were analyzed using a spectrometer (FAAS, PerkinElmer Model A300, Norwalk, USA) to determine the remaining concentrations of Hg(II) and Ni(II) in solution.

From the pH studies, the authors observed an optimum removal percentage of 99% for Hg(II) at a pH of 3.0 and 96% for Ni(II) at a pH 8.0. As shown in Figure 3b, there was an increase in the Q_e_ with increases in the original concentration. The data was also used to calculate the Q_max_ value of LDH/MOF by fitting it to the nonlinear isotherm models of Langmuir, Freundlich, and Redlich–Peterson. From their conclusions, the data fitted the Langmuir more than the other models with Q_max_ values of 509.8 mg g^−1^ for Hg(II) and 441.0 mg g^−1^ for Ni(II). Furthermore, the kinetic data, as presented in Figure 3c, showed that the LDH/MOF nanocomposite had fast kinetics for Hg(II) and Ni(II) and obeyed the Avrami kinetic model. Hence, AAS is appropriate for monitoring studies of a certain element. It is a fully automated procedure, and thus, a less labor-intensive method. The advantage is that this analytical method allows the determination of elements in very low mass concentrations (µg/L range), however, its disadvantage is the long analysis time per sample due to three or four replicates.

### 2.4. Ultraviolet-Visible Spectroscopy

The ultraviolet-visible (UV-Vis) spectroscopy is employed in studying the properties of samples by analyzing the amount of light they can absorb or reflect [47]. The light that is used in the instrument is in the wavelengths of UV and the visible region of the electromagnetic spectrum [47,48]. In principle, the light of a suitable wavelength is irradiated onto the molecule and absorbed by the π-electrons or non-bonding electrons within the molecule, and then detected and displayed as an absorbance peak. The absorbed wavelengths have energy associated with them and are responsible for the transition of electrons from the ground state to the excited state [49,50,51,52,53,54,55,56]. Depending on the composition of the sample that is being analyzed, quantitative and qualitative measurements can both be obtained by comparing the analyte with a reference sample. The absorbed energy provides information about the components that are present in the sample, and therefore, their concentrations can be determined. This energy is determined from Equation (2) using the energy difference between the lower and higher energy levels:

(2) E=hv

where *E*, *h*, and *v* represent the amount of energy absorbed, Planck constant, and the frequency, respectively. Then, Equation (2) is expanded into Equation (3), due to the wavelength associated with the light that is absorbed by molecules in spectroscopic studies [50,51]:

(3) $$E=\frac{hc}{\lambda }$$

where the speed of light is denoted by *c* and *λ* is the maximum wavelength of light absorbed by the analyte sample. The UV-Vis instrument estimates the intensity of absorbed light as a function of absorbance (*A*) or transmittance (*T*), which are related by Equation (4):

(4) A=−logT

The interaction between electromagnetic radiation and molecules can be defined using Beer’s law, which describes proportionality between the incident radiation and the concentration of the absorbing molecule with the rate of the monochromatic beam. Mathematically, Beer’s law is expressed as Equation (5) [50]:

(5) A=abc

where *A*, *c*, *a*, and *b* representing the absorbance, concentration of the analyte, absorptivity constant, and path length of a cell, respectively. Due to the unit of concentration being molar (M), Beer’s law is expressed as Equation (6):

(6) A=εbc

where *ε* denotes the molar absorptivity of the sample [47,48,49,50,51,52].

**Figure 4:**
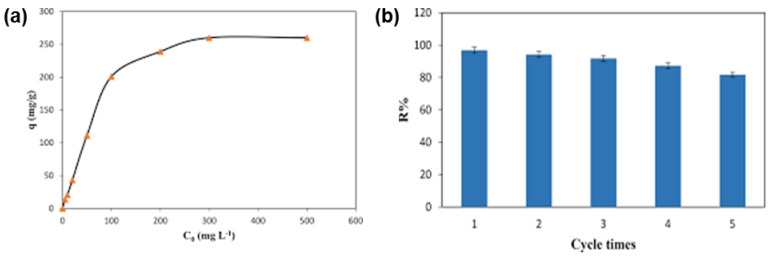
(**a**) The effect of initial Pd(II) ion concentration; (**b**) the reusability of UiO−66−Pyta for Pd(II) ions adsorption [57].

Daliran et al. [57] functionalized Zr-MOF with pyridyltriazol (Pyta) to selectively adsorb Pd(II) ions from an aqueous environment. For batch adsorption experiments, a 25 mL solution having 1 mg L^−1^ of Pd (II) at a pH of 4.5 was contacted with 0.01 g of UiO-66-Pyta for approximately 2−30 min. After adsorption, the authors separated the UiO-66-Pyta adsorbent from the Pd(II) ion solution by centrifuging at a speed of 5000 rpm and analyzed the supernatant with a UV−Vis instrument at a wavelength of 410 nm. Figure 4a presents the results obtained after studying the influence of the original concentration of Pd(II) ions on the Q_e_ value of UiO-66-Pyta. The data showed that Q_e_ increased as the original concentration increased, and the Q_max_ value was deduced from the Langmuir isotherm as 294.1 mg g^−1^.

The authors further conducted adsorption/desorption trials to explore the potential reusability of the UiO−66−Pyta composite for the adsorption of Pd(II) ions, and the results are shown in Figure 4b. The plotted data depicted that 96.9% was reduced to 81.7% after 5 consecutive cycles. The analysis conducted to determine the remaining concentrations of various metal ion pollutants in wastewater was shown to be effective using various detection techniques. Although some techniques have limited detection of other metal ion species, the obtained results display some potential utilization of the prepared MOF-based adsorbent material. Table 1 presents some of the studies wherein different metal ions have been analyzed by the chosen technique following adsorption with MOF composites. Therefore, there are several advantages of UV-Vis such as a broad area of applicability, high sensitivity with a limit of detection (LOD) in the mg/L range, high selectivity, and a simple and rapid automatic method. However, there are some disadvantages including time-intensive sample preparation and measuring procedure (binding to complexes, adjusting of the pH value, special extraction procedures) to obtain colored metal complexes which can be determined using UV-Vis interferences of other colored substances in the sample.

## 3. Characterization of MOF Composites for Heavy Metal Ions Adsorption

### 3.1. Physical Characterization

Physical methods are analytical techniques used to study and deduce information about the physical properties of compounds. These techniques assist in obtaining information about some of the phases that form part of the material structure, the potential surface reactivity looking at the area on the surface, as well as the functionality and possible geometry and atomic arrangements. They combine the fundamentals of both spectroscopic and microscopic techniques. Adsorption technology reveals information about the adsorption behavior of MOF composites.

#### 3.1.1. X-ray Diffraction

An X-ray diffraction (XRD) analytical instrument is mostly employed for determining the different phases of crystalline materials and obtaining data about the dimensions of the unit cell. The three major components of the XRD instrument are an X-ray tube, a sample holder, and an X-ray detector. The idea of this method is mainly grounded on the diffraction of light that is scattered by a periodic array of long-range order and results in the production of constructive interference at certain angles [67]. A beam of X-ray photons from the cathode ray tube passes through a slit where it is filtered to form monochromatic radiation that collimates to directly focus on the sample. Then, atomic or molecular crystals of powdered samples diffract the beam of X-ray photons, resulting in the scattering of photons in all directions [67,68,69]. When incident rays interact with an analyte, they result in a constructive interference obeying Bragg’s law which is presented by Equation (7) [70]:

(7) nλ=2dsinθ

where λ and *d* denote the incident light wavelength and spacing of diffracting planes, respectively. The angle associated with diffraction is represented by θ and *n* = 1 is an integer.

**Figure 5:**
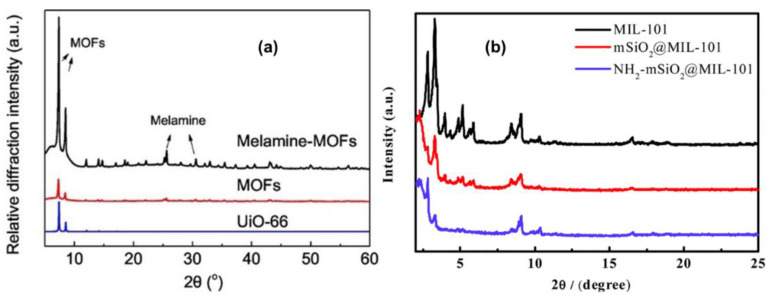
Diffractograms of: (**a**) MOFs and melamine-modified MOFs [73]; (**b**) pristine MIL-101(Cr), mSiO_2_@MIL-101(Cr), and NH_2_-mSiO_2_@MIL-101(Cr) [61].

XRD is commonly employed for identifying unknown compounds and measuring sample purity and crystallinity [67,68,69,71]. This technique is non-destructive to the sample and can be used for quantitative analysis, in which the data presentation includes two theta angles, peak intensity, and the amount of lattice constant. The data that are obtained for qualitative analysis include phase analysis, whereby the type of phase can be identified; phase composition; crystallite size; and orientation [68,72]. The XRD technique has been used by many researchers for the physical characterization of MOFs and MOF composites used in the adsorption of heavy metal ions. Moreover, after the adsorption process, the effects of the adsorbed metal ions on the phases and the crystallite size of MOF adsorbents have also been studied. From the obtained results, the crystallinity of the prepared materials have been deduced and, in some cases, the incorporation and modification with other functional components has been confirmed using this technique. For example, Yin and co-workers [73] synthesized UiO-66 MOF which they modified with melamine for the removal of Pb(II) ions. The diffraction patterns of UiO-66 and melamine-UiO-66 were obtained using an X-ray diffractometer (BRUKER AXS, D8 Advance), and the results are shown in Figure 5a. The patterns of the prepared UiO-66 and melamine-modified UiO-66 show some similarity with those of simulated UiO-66 from CCDC 837796. The authors concluded that post-modification with melamine did not disrupt the crystal structure of melamine-UiO-66. Moreover, the melamine-UiO-66 displayed higher peak intensities at two theta values of 7.4° and 8.5°, suggesting an increased degree of crystalline on the MOF structure. The melamine peaks were also observed at 2θ = 26° and 30° to further support the chemical interaction between melamine and the UiO-66. Quan et al. reported on NH_2_-mSiO_2_@MIL-101(Cr) composite for adsorbing Pb(II) and Cr(VI) ions which were characterized using a PANalytical Empyrean X-ray diffractometer operating at a scan rate of 5° min^−1^. As presented in Figure 5b, MIL-101(Cr) was prepared and modified with mSiO_2_ due to the appearance of a peak at 2θ = 2.5° on both the patterns of mSiO_2_@MIL-101(Cr) and NH_2_-mSiO_2_@ MIL-101(Cr). The diffraction patterns for MIL-101(Cr) are also visible in both composites indicating that the crystal phases are still intact, however, are less intense on NH_2_-mSiO_2_@ MIL-101(Cr) nanoadsorbent, confirming the effective grafting of amino groups.

**Figure 6:**
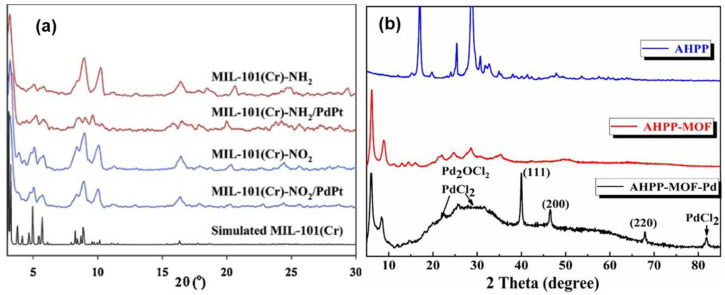
Diffractograms of: (**a**) MIL-101(Cr)-NH_2_, and MIL-101(Cr)-NO_2_, after the adsorption of Pd(II) and Pt(IV) ions [74]; (**b**) AHPP, AHPP-MOF, and AHPP-MOF-Pd [36].

Other researchers have used the XRD technique to partially understand the mechanism of adsorption taking place between MOFs and heavy metal ions. Lim and colleagues studied the removal of Pd(II) and Pt(IV) ions using MIL-101(Cr)-NH_2_ which was prepared by reducing MIL-101(Cr)-NO_2_ [74]. The patterns were measured between 3° < 2θ < 90° using a higher performance XRD, having a Cu-sealed tube of λ = 1.54178 Å. The XRD patterns for the prepared materials were obtained before and after metal ion adsorption, and are presented in Figure 6a. The authors concluded that the pristine MIL-101(Cr)-NH_2_ and MIL-101(Cr)-NO_2_ structures were stable in acidic media since they retained peaks that were identical to those of the simulated MIL-101(Cr) before and after the adsorption of Pd(II) and Pt(IV) ions.

Tang and his colleagues characterized AHPP-MOF-Pd after adsorbing Pd(II) ions and compared it with the diffraction peaks of AHPP and AHPP-MOF, as depicted in Figure 6b. According to their observations, the diffractograms of AHPP and AHPP-MOF were quite dissimilar. However, the peaks at 2θ = 6.0° and 8.09° for the AHPP-MOF corresponded to the conventional patterns of UiO-66 reported in the literature, indicating the successful combination of AHPP and ZrCl_4_. Furthermore, the diffraction peaks at 2θ = 22.3°, 26.7°, and 82.8° for AHPP-MOF-Pd were attributed to PdCl_2_ and Pd_2_OCl_2_. The authors also observed some peaks at 2θ = 40.061°, 46.506°, and 67.898° corresponding to the (111), (200), and (220) planes of palladium, respectively (JCPDS no. 65–2867). They observed that Pd(II) ions were interacting with some surface functional groups on the AHPP-MOF, which resulted in the formation of Pd compounds including metallic Pd. Although the XRD technique cannot provide information regarding the mechanism of interacting between MOFs and heavy metal ions, it is still of significant importance, since MOF structures have been shown to be very crystalline materials. Their successful composite formation with other compounds is most often confirmed by a reduction in crystallinity wherein some shift or broadening of diffraction peaks is observed as well as the appearance of new peaks. In addition, their interaction with heavy metal ions can also be confirmed by an increase or decrease in crystallinity. Hence, the XRD patterns of the MOFs composites after the adsorption of heavy metal ions should be obtained in order to confirm their interaction.

#### 3.1.2. Thermal Gravimetric Analysis

A thermal analysis is a study conducted using a group of analytical techniques combined to give important information about the physicochemical properties of a material as a function of temperature. Thermogravimetry is one of the techniques of thermal analysis used to study variations in the quantity and frequency of the weight of a sample against temperature and time in a controlled atmosphere such as purged nitrogen gas [75,76,77,78,79]. The main components of a TGA instrument are the furnace, the microbalance, the temperature controller, and a data acquisition system [80,81]. In principle, a sample with a mass of 2–20 mg is inserted into a pan (crucible) of suitable size, followed by setting the temperature variations according to an adapted temperature program. These may include isothermal ramp steps having various heating rates while measuring the temperature with thermocouples that are in contact with the crucible. In a TGA instrument, the sample holder for putting the crucible is connected to the microbalance (mass sensitive element) which is used for detecting the weight changes associated with the sample. Then, the sample holder system is heated with an electric furnace that can reach maximum temperatures of about 2000 °C. However, the quantity of heat needed depends on the specifications of material, the design of the furnace, as well as other components [76,78].

The weight change recorded as a function of time is in isothermal mode, whereas weight change captured as a function of temperature is in scanning mode. The non-isothermal mode is usually associated with a constant heating rate (β) which is caused by the linear change in temperature with time and is expressed by Equation (8):

(8) $$\beta =\frac{dT}{dt}$$

where *dT* and *dt* represent the change in temperature and change in time, respectively. The data obtained from TGA assist in deducing the type of reaction that can take place including decomposition, sublimation, vaporization, etc. Furthermore, gaseous products that escape during the chemical reaction, thermal stability of composites, and associated degradation mechanisms can be studied by this technique. Generally, a compound is regarded as being thermally stable if the TGA curve shows no change in sample weight. However, its disadvantage is the destruction of the sample as well as the restricted number of elements to be analyzed [79,81,82,83,84].

**Figure 7:**
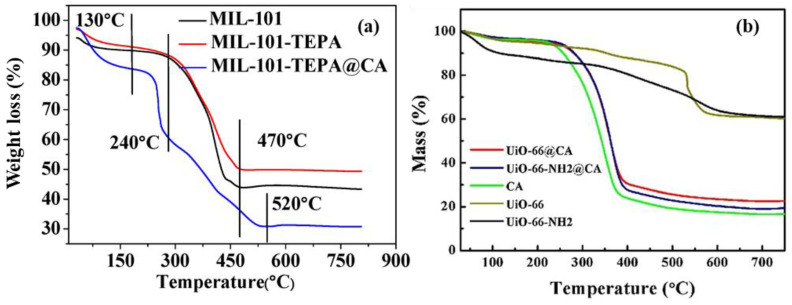
TGA curves of: (**a**) MIL-101(Cr), MIL-101(Cr)/TEPA, and MIL-101(Cr)/TEPA@CA [85]; (**b**) UiO-66, UiO-66-NH_2_, CA, UiO- 66@CA, and UiO-66-NH_2_@CA [86].

For the removal of heavy metal ions by MOF composites, authors have employed the TGA technique to examine the thermal stability of the synthesized materials. Depending on the MOF that one is working with and the type of modification taking place, the thermal stability of the resulting MOF composites (including nanocomposites) can either improve or degrade. The composite of MIL-101(Cr)/TEPA@CA (tetraethylenepentamine@calcium alginate) used in the adsorption of Pb(II) ions was synthesized by Wang et al. [85]. The obtained TGA curves for the composite and its precursors (MIL-101(Cr) and MIL-101(Cr)-TEPA) are shown in Figure 7a. The prepared materials demonstrated weight loss due to moisture at less than 130 °C with weight losses of 4.95%, 8.35%, and 10.10% for the MIL-101, MIL-101(Cr)-TEPA, and MIL-101(Cr)/TEPA@CA, respectively. The degradations occurring between 240–470 °C for MIL-101(Cr) and MIL-101(Cr)-TEPA corresponding to 45.21% and 39.96% weight loss were due to decomposition of the MIL-101 structure while converting to Cr_2_O_3_. For MIL-101(Cr)/TEPA@CA, the loss in weight increased by 49.64% at 240–520 °C and it included the conversion of cellulose aerogel (CA) into CaO. In another report, the thermal properties of the synthesized UiO-66-NH_2_@cellulose aerogel composite were studied by Lei et al. [86] through a comparison with the thermal stabilities of UiO-66, UiO-66-NH_2_, and CA materials. The thermograms were obtained from a TGA, TG 209 F1, Germany instrument that operated at 20 °C min^−1^ heating rate and 30–700 °C under N_2_ atmosphere. As presented in Figure 7b, the CA was less thermally stable than MOF and showed maximum decomposition at 332.2 °C, whereas the value increased by 26.2 °C for UiO-66@CA and 26.7 °C for the UiO-66-NH_2_@CA composite. The prepared UiO-66- NH_2_@CA composite with improved thermal stability was utilized in the adsorption of Pb(II) ions from simulated wastewater. It was observed in TGA that the processes occurring during heating of the samples directly coincided with temperature. This suggested that the adsorption of heavy metals occurred on the surface of the MOF structure. TGA can reveal the chemical stability of MOFs after adsorption which strongly depends on the possibility of preserving their initial structure. The exchange of heavy metals and guest anions from the aqueous solution to the MOF surfaces can be observed by changes in thermal stability. The weight loss from TGA can be used to estimate the amount of heavy metals on the surface of MOF materials by determining changes in the mass of the MOF composites before and after adsorption.

#### 3.1.3. Differential Scanning Calorimetry (DSC)

Differential scanning calorimetry is another thermal analytical technique which was developed in 1962 by Watson and O’Neill and commercialized in 1963 [87]. This thermal analytical technique uses the same operating conditions as TGA and is sometimes coupled together in a system referred to as simultaneous thermal analysis (STA) [78]. DSC is based on the measurements of heat flow between a sample and inert reference materials as a function of temperature, wherein the changes in heat capacity and endothermic and exothermic activities occurring on a sample can be determined. It is a powerful and rapid method for providing qualitative and quantitative data concerning the physicochemical phase transitions experienced by materials when exposed to elevated temperatures (from 100 to 1800 K) [80,81,88,89,90,91,92,93]. The three basic phases that can occur when an amorphous substance is being exposed to heat are as follows: (a) glass transition, in this phase change, the structure of an amorphous substance changes from a moderately hard state to a rubbery state and it is reversible, and the glass transition temperature provides information concerned with the stability of the glassy or amorphous state; (b) crystallization, this irreversible (two-steps) phase change entails assembling the disordered structures through nucleation and growth processes to form crystalline structures through an exothermic process; (c) melting, in this transition, a single step endothermic process occurs when the crystalline lattice becomes broken down into a disordered state from solid to liquid in a single step [87].

**Figure 8:**
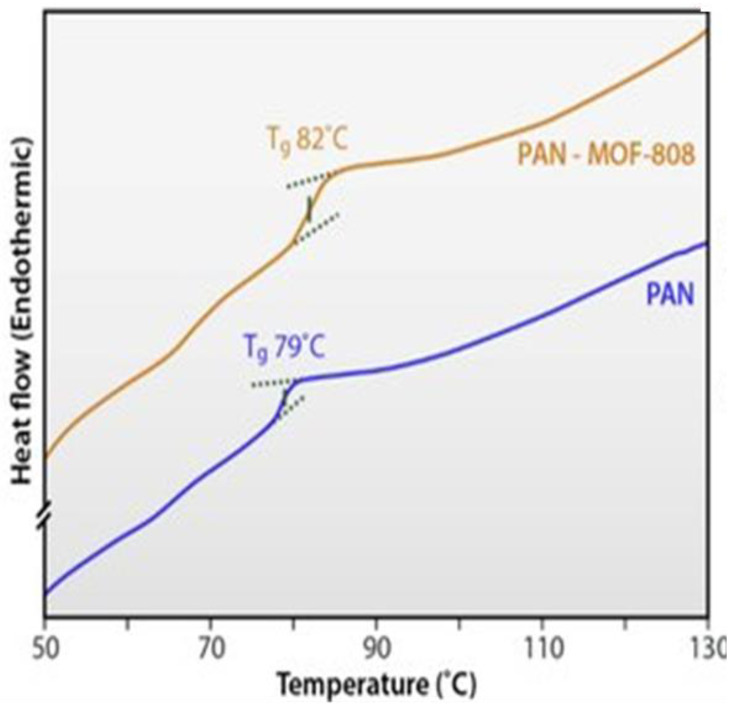
DSC thermogram of PAN and PAN@MOF-808 nanomembranes [94].

Efome et al. [94] synthesized polyacrylonitrile (PAN) nanofiber-supported Zr-based MOF-808 via co-electrospinning for the adsorption of Cd^2+^ and Zn^2+^ from an aqueous environment. The glass transition temperatures (Tg) of the PAN and PAN@MOF, as obtained from a TA Instruments DSC Q2000 V24.11 Build 124, are presented in Figure 8. The results were obtained by annealing approximately 5 mg of the nanomaterials for 10 min at 150 °C, followed by quenching to 25 °C for another 10 min, and then the heating rate for Tg measurements was 5 °C min^−1^. The DSC thermogram shows that the Tg of PAN@MOF-808 increases slightly by 3 °C, to 82 °C as compared with the initial Tg value of 79 °C for PAN. The shift is attributed to the restricted chain movement on the PAN polymer brought about by its interaction with MOF-808. DSC is similar to TGA, it can also be used to monitor the adsorption mechanism of heavy metals by looking at the change in heat flow as a function of the sample temperature. The transition of heavy metal ions or guest anions from aqueous solution to the surface MOF composites can be observed by changes in heat flow corresponding to the endothermic and exothermic peaks.

#### 3.1.4. Brunauer, Emmett, and Teller Method

The measurement of the surface areas of materials is one of the studies that is conducted to deduce some of the properties of materials. The most commonly used method, developed by Brunauer, Emmett, and Teller in 1938, is the BET theory [95]. The main working principle of the BET analytical technique is associated with the amount of gas adsorbed onto the surface of materials. The types of interactions that can occur between an adsorbate and the adsorbent are physisorption (via van der Waals) and chemisorption (via chemical reaction) [95,96]. After the adsorbate–adsorbent interaction, the reaction ultimately reaches equilibrium at a particular constant temperature and relative vapor pressures denoted as *P/P_o_*. The quantity of the adsorbed gas is determined and the result is used to produce an adsorption isotherm. The total gas captured has a proportional relationship with the external and internal surfaces of the adsorbent material [96,97]. The BET theory can be derived similarly to the Langmuir theory (assumes monolayer adsorption between gaseous atoms and the adsorbent surface). However, multilayer adsorption can occur if the surface temperature of the adsorbent is less than the critical temperature of the adsorbate (gas molecules). In this process, many layers of adsorbed gas molecules form; however, some of them are not in contact with the adsorbent surface layers. The BET theory assumes multilayer adsorption where all layers are in equilibrium and atoms on the lower layers serve as adsorption sites for atoms on the above layers and the BET equation is expressed by the following Equation (9):

(9) $$\frac{P/Po}{n\left(1-\frac{P}{Po}\right)}=\frac{1}{n_{m}C}+\frac{C-1}{n_{m}C}\left(\frac{P}{Po}\right)$$

where *n* and *n_m_* denote the specific amount and monolayer capacity of the gas adsorbed, respectively. *C* represents the BET constant relating to the monolayer adsorption energy and it can be used to determine the shape of the isotherm [95,96,98,99,100,101,102,103].

The BET isotherms can form six different curves. Type I isotherms, which are reversible, have two patterns and are obtained from microporous solids having micropore widths that are below ≈1 nm. Type II isotherms are common for compounds that are nonporous or macroporous and are also reversible. Type III isotherms are achieved when the adsorbent−adsorbate interactions are weak and the monolayer surface coverage data are not given. Type IV isotherms have two patterns that are related to the width of the pores. However, type IV isotherms are reduced to type VI isotherms if the size of the width is higher than the critical width. Type V isotherms are observed at low *P/P_o_* ranges and result from weak interactions between the adsorbent and adsorbate. Type VI isotherms are usually obtained over multilayer adsorption on substances having extremely uniform nonporous surfaces. The stepwise-shaped curves depend on the material, gas, and temperature [95,101,103,104].

**Figure 9:**
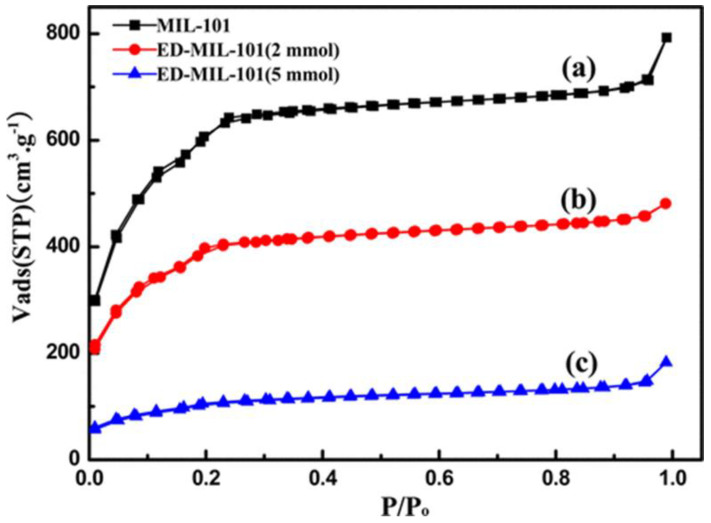
BET isotherms of: (**a**) MIL−101(Cr); (**b**) ED−MIL−101(Cr) 2 mmol; (**c**) ED−MIL−101(Cr) 5 mmol [105].

The BET technique has been used to deduce the surface area, pore volume, and pore diameter of MOFs and MOF composites. From the reported literature, MOFs have been described as highly porous materials with very high surface areas that can reach over 3000 m^2^ g^−1^. Furthermore, the pore volume and pore diameter that correspond to the specific surface areas of MOFs can be determined. The surface areas and pore volumes of MOFs both enable MOFs to be good host that can accommodate a variety of guest molecules. Since the adsorption of heavy metal ions by MOF composites is a surface phenomenon, the BET technique has been used to confirm the interactions of MOF materials with the heavy metal ions. During adsorption, these heavy metal ions penetrate into the pores of MOF composites resulting in a reduction in the surface area and subsequently the pore volume decreases indicating that there are some molecules occupying their space. Such information can be obtained by an analysis of an MOF composite after the adsorption. In a study reported by Luo and his colleagues [105], MIL−101(Cr) was prepared and functionalized with ethylenediamine (ED) for the adsorption of Pb(II) ions from an aqueous solution. The BET surface areas of MIL−101(Cr) and ED−MIL−101(Cr) are shown in Figure 9a. The isotherm curves demonstrate a type I behavior and the obtained BET surface area of 2290 m^2^ g^−1^ for MIL−101(Cr) decreases significantly after grafting with 2 and 5 mmol of ED to 1270 and 347 m^2^ g^−1^, respectively. Furthermore, the corresponding pore volume of 1.4 cm^3^ g^−1^ for MIL−101(Cr) shows the same trend of decreasing to 0.74 cm^3^ g^−1^ for 2 mmol ED and 0.28 cm^3^ g^−1^ for 5 mmol. The reduction in the porosity is attributed to the occupation of some pores on the MIL−101(Cr) by the ED moieties after surface modification, and thereby, preventing the adsorption of N_2_ molecules. In another study, the porous nature of the prepared AHPP-MOF composite was deduced from ASAP-2020 plus, Micromeritics, USA.

**Figure 10:**
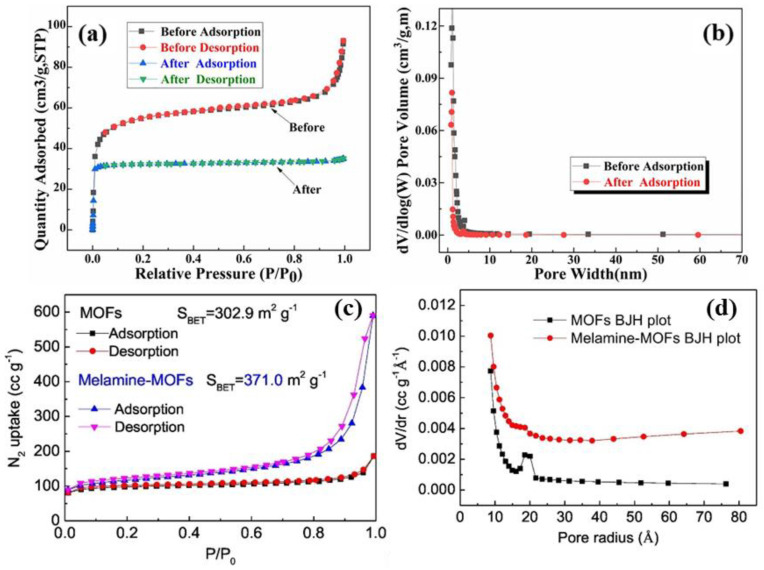
BET curves of: (**a**) AHPP−MOF and Pd@AHPP−MOF; (**b**) pore width distribution curve (before and after) adsorption [36]; (**c**) the MOFs and melamine−modified MOFs; (**d**) pore size distribution BJH plot [73].

The N_2_ adsorption-desorption isotherm curves before and after Pd(II) adsorption are represented in Figure 10a and the corresponding pore volumes are shown in Figure 10b. From the BET isotherms of AHPP-MOF, it can be noted that the type IV behavior is the prevailing curve. This characteristic hysteresis loop describes the porous nature of the AHPP-MOF with the calculated specific surface area and pore volume of 180.29 m^2^ g^−1^ and 0.09 cm^3^ g^−1^ [36]. After the adsorption of Pd(II) ions, both the specific BET surface area and pore volume decreased, confirming the occupation of the surface pores by the Pd(II). Yin and co-workers [73] reported that the functionalization of MOF materials with melamine increased the BET surface area of the final composite of melamine-modified MOFs. As shown in Figure 10c, the obtained type I shape of the N_2_ adsorption-desorption isotherm curve confirmed the microporous surfaces. However, the melamine-modified MOFs curve demonstrated a type IV isotherm shape which suggested the mesoporous natures of the functionalized MOF surfaces. Furthermore, the specific BET surface areas of the MOFs increased from 302.9 to 371.0 m^2^ g^−1^ for the melamine-modified MOF composites and the behavior was supported by pore size distribution, as presented in Figure 10d. The results showed that MOFs had a narrow pore size distribution of about 20 Å, whereas the melamine-modified MOFs displayed a fairly larger pore size distribution with mesoporous architectures [73].

### 3.2. Microscopic Characterization

Microscopy is another powerful technique that is employed to study the morphological structures of prepared materials. The technique acquires information about the material at a microscopic level wherein different images relating to the structures of materials before and after functionalization are obtained. Furthermore, these techniques can be coupled with various detectors to reveal the elements that are present in the structures as well as their distribution on the surface of the composites.

#### 3.2.1. Scanning Electron Microscopy—Energy Dispersive Spectroscopy

The most powerful and versatile analytical technique used for studying and analyzing the morphological micro-, nanostructure, and chemical composition of materials is scanning electron microscopy (SEM) [106]. This technique involves generating a beam of electrons that have an energy of approximately 40 keV and is bombarded on the sample of interest. This beam interacts with the surface of the analyte by scanning it using scan coils [107,108,109]. This phenomenon results in excitation of the electrons on the surface and subsequently causes elastic and inelastic collisions until the electrons possess enough energy to escape. This interaction is followed by scanning of electrons along parallel lines, which emits various signals due to Auger electrons, secondary electrons, backscattered electrons, X-rays, and photons. Then, the particles that originate from the sample are collected by various detectors and produce an image or information about the surface of the analyte [81,106,110,111,112,113,114]. The SEM instrument is mostly coupled with the energy-dispersive X-ray spectroscopy (EDS) to attain qualitative data about the composition of the sample of interest. EDS works by detecting and “counting” the X-rays generated by the emitted electron beam. When an incident beam of electrons strikes the surface of an analyte, it causes the excitation of inner shell electrons which leaves vacant sites that are filled by electrons in the outer shell. This transition is accompanied by the release of X-ray energy that is signified by the differences in energy amongst the inner and outer shell electrons. The X-ray photons characterize all the elements in the periodic table except H, He, and Li. The elements that are present as major constituents can be identified and quantified [114,115,116,117,118].

**Figure 11:**
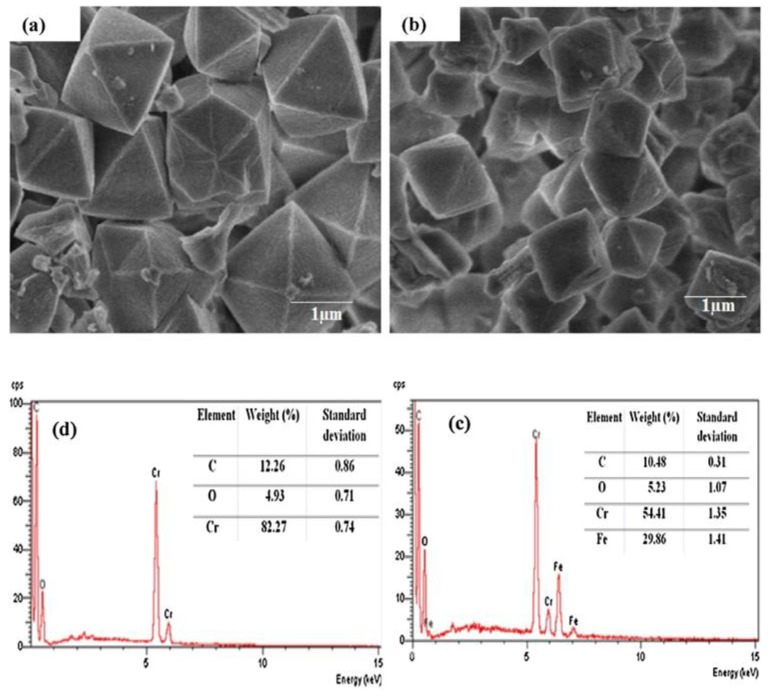
SEM images of: (**a**,**b**) of MIL−101(Cr); (**b**) Fe–MIL−101. EDS analysis of: (**c**) MIL−101(Cr); (**d**) Fe–MIL−101 [120].

The SEM-EDS technique has been employed for the morphological and elemental studies of MOFs and MOF composites/nanocomposites. This instrument provides two-dimensional images of higher resolution that reveal the geometry of a sample as well as spatial variations. Furthermore, the data can be used to acquire evidence regarding the external morphology, dispersion, and various phases of a sample [119]. Thanh et al. [120] synthesized and compared two different MOFs (i.e., MIL−101(Cr) and Fe–MIL−101) for the adsorption of Pb(II) from an aqueous solution. The obtained SEM images and EDS analysis, as shown in Figure 11a, indicated an octahedral geometry with smooth facets for MIL−101(Cr) and the elemental analysis (see Figure 11d) confirmed the presence of Cr, O, and C which were the major constituents of MIL-101. The image of Fe–MIL−101, as represented in Figure 11b, displayed irregular shapes mixed with octahedral structures. Further support from an elemental analysis, as shown in Figure 11c, confirmed the elemental composition of the prepared MOF with the major constituents being Fe, Cr, O, and C.

**Figure 12:**
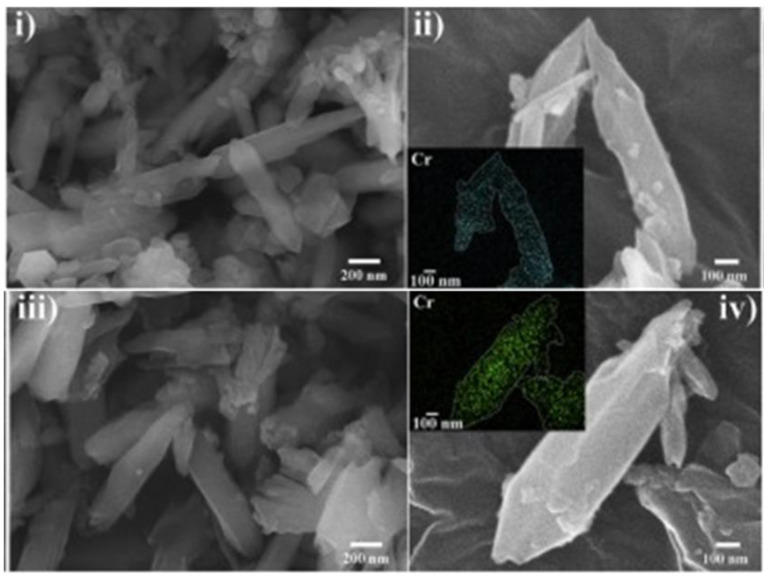
FE-SEM images of: (**i**,**ii**) MIL−101(Cr)−NH_2_; (**iii**,**iv**) MIL−101(Cr) −NO_2_ (insets show chromium elemental mapping images) [74].

**Figure 13:**
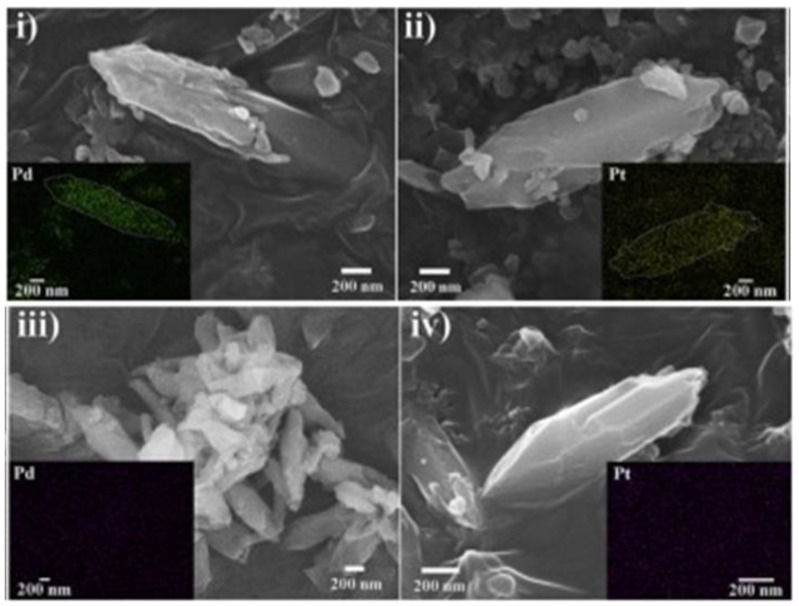
SEM and elemental mapping images of MIL−101(Cr)−NH_2_ after: (**i**) Pd loading; (**ii**) Pt loading. MIL−101(Cr)−NO_2_ after: (**iii**) Pd loading; (**iv**) Pt loading [74].

In another study, Lim et al. [74] described the synthesis of MIL−101(Cr) −NO_2_ using CrCl_3_ as a source of Cr and reduced it to MIL−101(Cr) −NH_2_ using SnCl_2_ for removing Pd(II) and platinum Pt(VI) in acidic solutions. The SEM images, as shown in Figure 12, revealed prismatic crystals with a diameter and length of approximately 150 nm and 1000 nm, respectively, for both the MIL−101(Cr) −NH_2_ and MIL−101(Cr) −NO_2_. Furthermore, the inset elemental mapping images showed the even distribution of Cr on the surface of the MIL−101(Cr)−NH_2_ and MIL−101(Cr)−NO_2_ [74]. For example, the images obtained after the adsorption of both the Pd (II) and Pt (IV) which were supported by elemental mapping (see insets) are shown in Figure 13i–iv. For the PGM−loaded MIL−101(Cr) −NH_2_ (Figure 13i,ii), an even distribution of the prismatic crystals particles of the composite was observed. This was attributed to the high adsorption capacity of MIL−101(Cr) −NH_2_ towards Pd(II) and Pt(IV). Conversely, the low adsorption capacity of MIL−101(Cr)−NO_2_ showed the inconspicuous distribution of Pd(II) and Pt(IV) on the elemental mapping images (Figure 13iii,iv).

#### 3.2.2. Transmission Electron Microscopy—Energy Dispersive X-ray Spectroscopy

**Figure 14:**
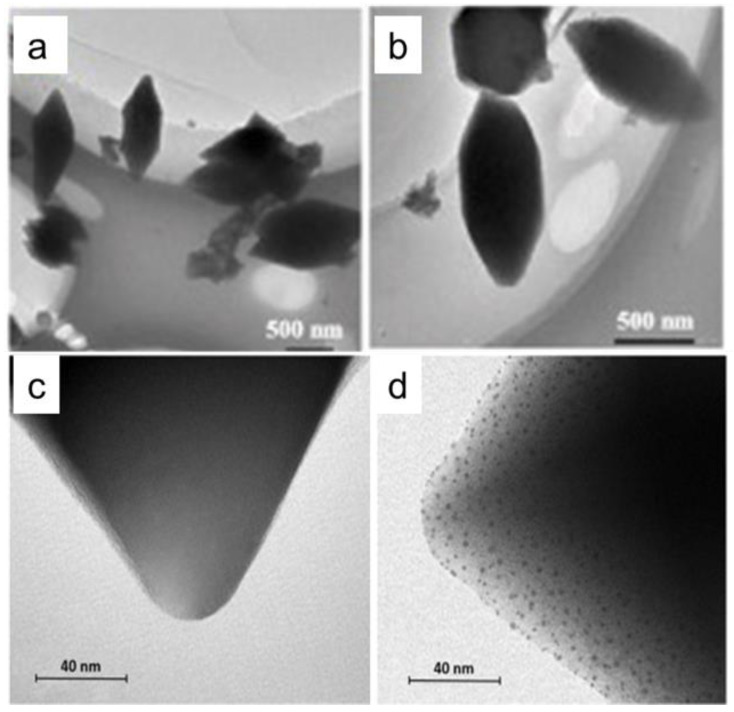
TEM images of: (**a**) MIL−101(Cr) NH_2_; (**b**) MIL−101(Cr) [127]; (**c**) MIL−101(Cr); (**d**) 12% Cu@MIL-101(Cr) crystal [128].

**Figure 15:**
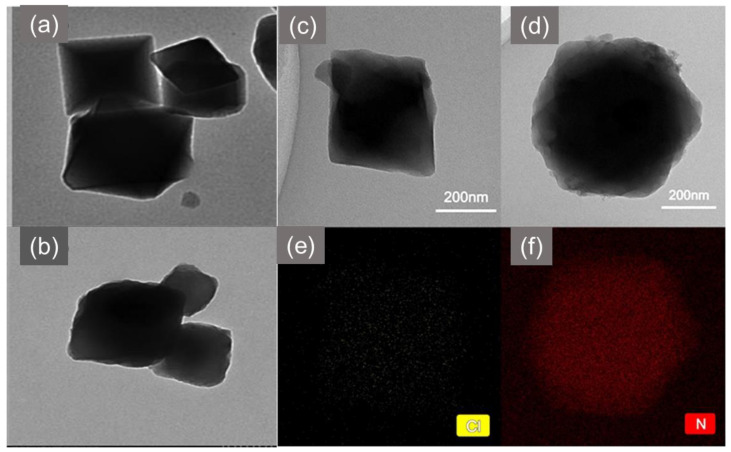
TEM images of: (**a**) MIL−101 and (**b**) ED−MIL−101 (5 mmol) [105]; (c) MIL−101−CM, (**d**) MIL−101−AO andEDS mapping images of: (**e**) MIL−101−CM and (**f**) MIL−101−AO [129].

Transmission electron microscopy (TEM) is a highly effective analytical technique used to study the internal surface morphologies of various materials. Its working principle is similar to that of a SEM instrument and can also be coupled with various detectors to attain data associated with the structure of the analytes. The difference with the TEM instrument is that the energies of the incident beam of electrons are much higher as compared with the SEM instrument [112,121,122]. This beam of primary/incident electrons, with energies between 80 and 300 keV, passes through lenses where it is filtered and focussed on the sample of interest. The beam of electrons penetrates into the sample and inelastically collides with the inner atoms, resulting in the emission of secondary electrons and X-rays. The emitted electrons that are scattered through smaller angles in all directions are limited and focussed onto the projector lens by the objective aperture. Subsequently, the image is collected onto the detector screen and its contrast is enhanced by altering the voltage from the gun [123,124,125]. High-quality images with more information about a sample are formed due to the fact that the fast-moving electrons having shorter wavelengths. Many researchers have used TEM coupled with an energy dispersive X-ray spectroscopy (EDX) detector to study and obtain evidence relating to the structure, texture, shape, and size of MOFs and their composites [124,126]. For example, Lv and his colleagues [127] compared the internal morphologies of prepared MIL−101(Cr) and MIL−101(Cr)−NH_2_ which were synthesized via the solvothermal method with the use of dimethylformamide (DMF). The MIL−101(Cr) was functionalized with amino functional groups to form MIL−101(Cr)−NH_2_ which was employed for removing Pb, Cu, and Fe metal ions. As shown in Figure 14a, the synthesized MIL−101 exhibited a hexagonal prismatic structure which remained intact after the introduction of amino groups, as depicted by Figure 14b. Abedidni et al. [128] hydrothermally synthesized MIL−101 followed by post-modification with cuprous oxide nanoparticles (Cu_2_O) for the adsorptive equilibria and kinetics separation of propylene. The obtained TEM images of the MIL−101(Cr) and 12%Cu@MIL−101(Cr) are presented in Figure 14c,d, respectively. The evidence revealed that Cu_2_O nanoparticles with sizes ranging between 1 and 3 nm were homogenously dispersed within the MIL−101(Cr) pores, and confirmed the successful reduction of the metal precursor into nanoparticles. In another study conducted by Luo and co-workers, MIL−101(Cr) with improved adsorption capacity for Pb(II) ions was prepared by PSM using ethylenediamine (ED) in anhydrous toluene. The authors compared the TEM images of MIL−101(Cr) before and after modification, as represented in Figure 15a,b. The MIL−101(Cr) showed octahedral structures with smooth surface, however, after the incorporation of ED, the surface became rougher [105].

In contrast, a study on the post-synthetic modification (PSM) of MIL−101 with amidoxime (AO) for removing uranium ions from seawater, which was conducted by Liu et al. [129], demonstrated a significant effect on the structure of MIL-101. The authors initially choloromethylated MIL−101 and obtained an octahedral structure with a uniform dispersion of the Cl element, as shown by TEM-EDS images of MIL−101−CM in Figure 15c,e. This was followed by grafting with diaminomaleonitrile before introducing amidoxime which resulted in an almost spherical morphology (Figure 15d). The significant change in the structure indicated the successful incorporation of AO and was further supported by the TEM-EDS mapping image which depicted a uniformly dense dispersion of N element from the AO on the MIL−101−AO (Figure 15f).

### 3.3. Spectroscopic Characterization

#### 3.3.1. Fourier Transform Infrared Spectroscopy

The main working principle of Fourier transform infrared spectroscopy (FTIR) is based on the interactions of molecules/compounds with light in the infrared region of the electromagnetic spectrum. This analytical technique offers the opportunity to obtain information about the functional groups that are present in a sample (solid, liquid, or gas), as well as the possibility of understanding the molecular bonds that exist between matter [81,130]. In IR spectrophotometry, a beam of infrared radiation emitted from the source is passed through an interferometer and is spectrally encoded, creating an interferogram (i.e., constructive and destructive interferences). This is followed by the light interacting with the sample where specific frequencies are absorbed by the sample. The resulting vibrational frequencies due to the bonds that are present in molecules are detected and calculated in terms of wavenumbers ranging from 4000 to 400 cm^−1^ [81,111,130,131,132,133,134,135]. On the FTIR spectra, there are four regions where the different types of bonds can be clearly analyzed. The first region, from 2500 to 4000 cm^−1^, corresponds to single O-H, C-H, and N-H bonds. It is followed by triple bonds which are found in the range between 2000–2500 cm^−1^. The middle region, with the wavenumber range from 1500 to 2000 cm^−1^, corresponds to detection of double bonds; the last area, below 1500 cm^−1^, is the fingerprint region where the vibrations of carbon single bonds between the atoms produce overlapping bands [131]. The FTIR technique has been widely used to deduce the functional groups that form as part of the MOF structure as well as to understand its interaction with other materials after composite and nanocomposite formation for the removal of heavy metal ions from wastewater [4,59,62,136,137].

**Figure 16:**
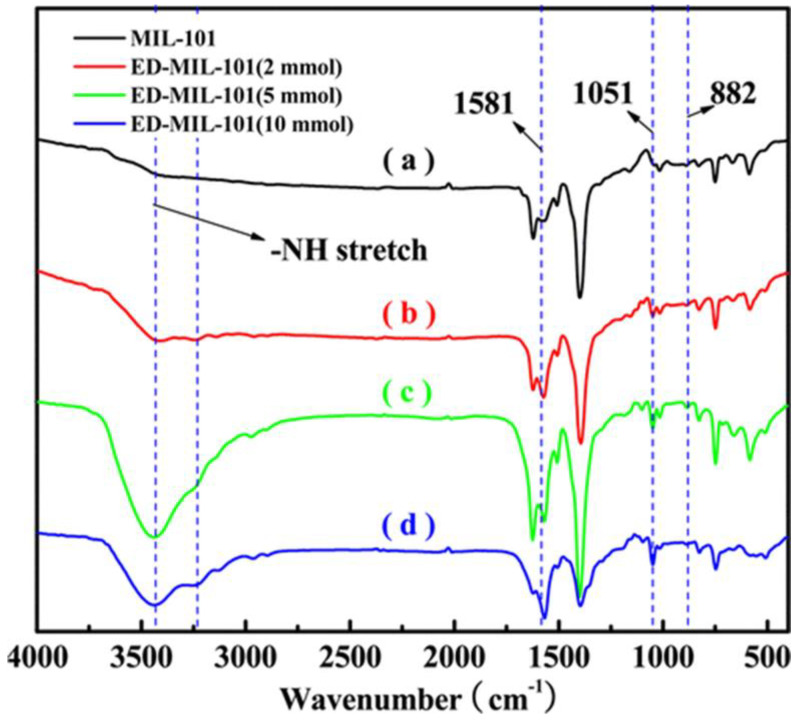
FTIR spectra of: (**a**) MIL−101(Cr); (**b**) ED−MIL−101(Cr) 2 mmol; (**c**) ED−MIL−101(Cr) 5 mmol; (**d**) ED−MIL−101(Cr) 10 mmol [105].

For example, Luo et al. [105] synthesized MIL−101(Cr) which was functionalized with ED for the adsorption of Pb(II) ions from wastewater. The amount of ED was varied to achieve the optimum removal of Pb(II), and the results are shown in Figure 16. The IR spectra for the functionalized ED−MIL−101 demonstrated peaks at 1581, 1051, and 882 cm^−1^ which were attributed to the N−H plane stretching, C−N bond stretching, and −NH_2_ stretching, respectively. Furthermore, the broad band between 3434 and 3231 cm^−1^, which was attributed to the −NH stretching, showed an increase in the intensity with an increase in the amount (2, 5, and 10 mmol) of ED grafted.

**Figure 17:**
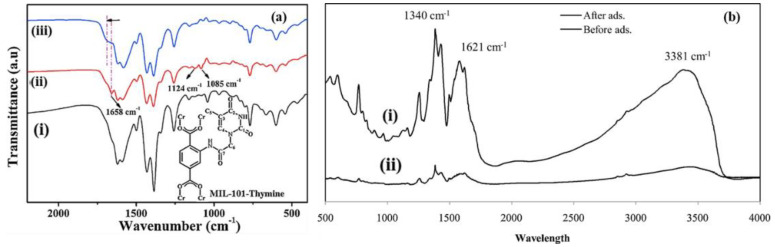
(**a**) FT-IR spectra of (i) MIL−101−NH_2_, (ii) MIL−101−thymine, and (iii) Hg-loaded sample of MIL−101−thymine [138]; (**b**) amino-functionalized MIL−101(Cr) before and after Cr(VI) adsorption [139].

In another study, MIL-101-NH_2_ was functionalized with thymine for the adsorption of Hg(II) ions from wastewater [138]. The IR spectrum of the resulting composite after adsorption was also obtained and compared with MIL−101−thymine, as presented in Figure 17a(i). The authors numbered the carbon atoms on MIL-101-thymine for easy interpretation. The spectrum of MIL−101−NH_2_ revealed peaks at 1658 cm^−1^ which were attributed to the stretching vibrations of C_2_=O and C_7_=O. Moreover, stretching vibration absorption bands of C_3_–C_5_ and C_6_–N were observed at 1124 and 1085 cm^−1^, respectively. All these distinct peaks were detected on the IR spectrum of MIL−101-thymine (Figure 17a(ii)). However, after the adsorption of Hg(II) ions, the absorption peaks of C_2_=O and C_7_=O became broad with a shift to higher wavenumbers (Figure 17a(iii)). Jalayeri and co-workers reported on the IR spectra of MIL−101(Cr) which was functionalized with amine moieties via the organic linker (AFMIL) for the removal of hexavalent chromium from an aqueous solution [139]. The spectrum for the AFMIL, as presented in Figure 17b(i), showed a peak at 3381 cm^−1^ which was attributed to stretching vibrations of the amino moieties. Furthermore, the N–H bending vibrations and C–N stretching of the aromatic amines were observed at 1621 and 1340 cm^−1^, respectively. After the adsorption of hexavalent Cr, the IR spectrum (Figure 17b(ii)) showed some reduction in peak intensity as well as a slight shift to higher wavenumbers, which confirmed the chemical interaction between Cr and amino moieties. In addition, the IR is also one of the characterization techniques that can be utilized to understand the mechanism of heavy metal adsorption using MOFs. Peng et al. [24] showed the IR spectra of MOF−808−EDTA before and after for La^3+^, Hg^2+^, and Pb^2+^ adsorption and revealed the shift of C-N vibration mode from 1214 cm^−1^ to 1220 cm^−1^, 1242 cm^−1^, and 1250 cm^−1^. The shift in bands indicated the strong interaction between heavy metal ions and the grafting of the EDTA functional groups on the framework, which resulted in the chelate complex after adsorption.

#### 3.3.2. X-ray Photoelectron Spectroscopy

X-ray photoelectron spectroscopy (XPS) is an analytical technique that uses the photoelectric effect to obtain information about the chemical nature of a material at the atomic and molecular levels [81,113]. This surface-sensitive method involves the irradiation of an X-ray beam onto the surface of a sample. The atoms in the sample absorb the incident light and result in the emission of core electrons of which their kinetic energy (KE) is measured according to Equation (10):

hν = BE + KE + Φ_spec_ (10)

where hv denotes the energy of the X-ray and Φ_spec_ is the spectrometer work function.

The rearranged form of Equation (11) is used to calculate the binding energy (BE):

BE = hv − KE − Φ_spec_ (11)

The XPS spectrum is obtained as a function of the number of photoelectrons spotted against the BE. The photoelectron peaks are extracted from the orbital of the elements they were emitted from, and their corresponding BE permit their recognition. The peak intensities generated by the photoelectrons are directly proportional to the concentrations of the elements and can be used for their quantification. The XPS techniques can be used quantitatively to further reveal data regarding the empirical formula, the electronic and chemical states of elements (excluding hydrogen and helium) found within a sample, as well as their interactions with metal centers [81,111,113,140,141,142]. In the adsorption of heavy metal ions from wastewater by MOF materials, this technique has been widely used to study and understand the type of interactions taking place as well as to support the deduced adsorption mechanism.

**Figure 18:**
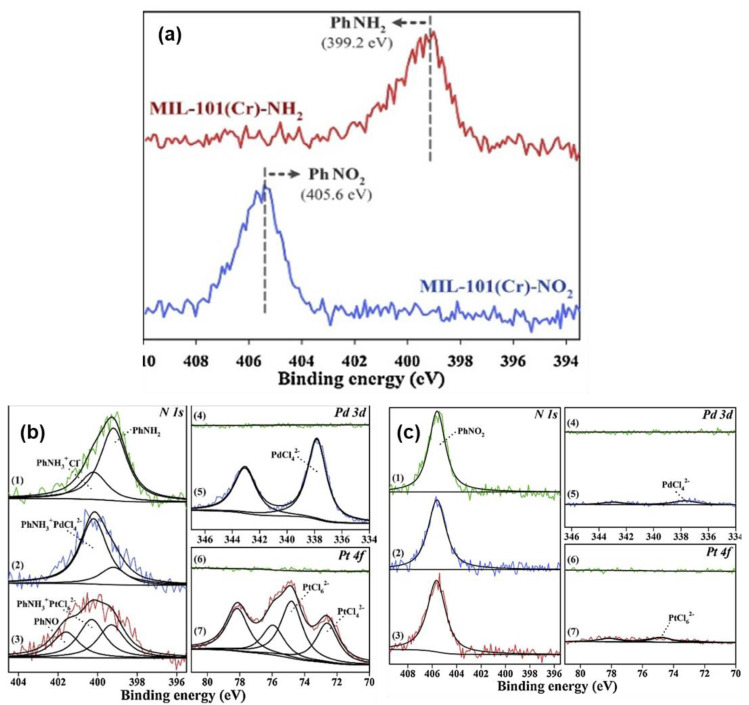
(**a**) XPS for N 1s spectra of MIL−101(Cr)−NH_2_ and MIL−101(Cr)−NO_2_; (**b**) MIL−101(Cr)−NH_2_ after Pd loading (2) Pd and Pt-loading (3), Pd 3d and Pt 4f of pristine ((4) and (6)), and metal-loaded MIL−101(Cr)−NH_2_ ((5) and (7)); (**c**) XPS for N 1s of MIL−101(Cr)−NO_2_ (1) after Pd loading (2), Pt loading (3), XPS for Pd 3d and Pt 4f of MIL−101(Cr)−NO_2_ ((4) and (6)), and after metal-loading ((5) and (7)) [74].

For example, Lim and co-workers studied the interactions of MIL−101(Cr)−NO_2_ and MIL-101(Cr)-NH_2_ with Pd(II) and Pt(IV) ions in order to understand the mechanism of adsorption [74]. Initially, the XPS N 1s spectra were obtained to confirm the reduction of the NO_2_ to NH_2_, as represented in Figure 18a. The MIL−101(Cr)−NO_2_ spectrum showed a peak at 405.6 eV, which was attributed to the nitro functional group that was attached to the phenyl ring (PhNO_2_). However, the peak diminished upon the reduction process, as the spectrum for MIL−101(Cr)−NH_2_ revealed a new peak at 399.2 eV which was ascribed to the amino group that was attached to the phenyl ring. After the adsorption of Pd and Pt ions, the authors obtained and compared the XPS N 1s spectra for metal-loaded MIL−101(Cr)−NH_2_ with the pristine, as shown in Figure 18b. The spectrum in (Figure 18b(1)) revealed two intense peaks at 399.2 and 400.2 eV which were attributed to the N in PhNH_2_ and PhNH_3_^+^ (interaction with H^+^ and Cl^−^ that remained after the synthesis process). As compared with the Pd-loaded MIL−101(Cr)−NH_2_ (Figure 18b(2)), there was an increase in intensity of the peak at 400.2 eV and a decrease in the intensity of the peak at 399.2 due to the electrostatic interaction of PhNH_3_^+^ with the [PdCl4]^2−^. Similar trends were also observed on the spectrum (Figure 18b(3)) of Pt-loaded MIL−101(Cr)−NH_2_, where the electrostatic interaction was between PhNH_3_^+^ and [PtCl_6_]^2−^. In addition, the appearance of a new peak at 401.9 eV was also observed, which was due to the partial oxidation of PhNH_2_ to PhNO_2_. Furthermore, the Pd 3d spectrum (Figure 18b(5)) revealed a peak at 337.8 eV corresponding to the [PdCl_4_]^2−^ adsorbed on the MIL−101(Cr)−NH_2_. For the Pt 4f spectrum (Figure 18b(7)), two major peaks of (4f _7/2_) attributed to the [PtCl_6_]^2−^ and [PtCl_4_]^2−^ were observed at 74.8 and 72.6 eV, respectively. Conversely, the MIL−101(Cr)−NO_2_ N 1s spectrum, as shown in Figure 18c(1–3), showed no significant effects on the adsorption of both the Pd and the Pt, as the peak at 405.6 eV showed no adverse effects which was further supported by the low intensity observed on the Pd 3d and Pt 4f spectra. Moreover, the excellent performance of MOF composites for heavy metal adsorption could be traceable to the electrostatic or strong chelation. The wide-scan XPS spectra indicated that the metal ions were adsorbed in the pores of MOF. In the case of functionalized MOF, the N 1s core level was shifted to higher binding energy upon metal loading (399.8 eV, 399.5 eV, and 399.9 eV for La^3+^@MOF−808−EDTA, Hg^2+^@MOF−808−EDTA, and Pb^2+^@MOF−808−EDTA, respectively) as compared with the as-synthesized MOF−808−EDTA (399.2 eV) [26]. This showed that the valence of N in EDTA was changed because of the interaction with the guest metal ions.

## 4. Conclusions

Some positive aspects of adsorption technology have been demonstrated in the remediation of wastewater containing heavy metal ions. Adsorption technology has been proven to be efficient in removing pollutants from contaminated water owing to its ease of operation and low cost. MOF polymers have shown some potential activity as adsorbents for the adsorption of metal ion pollutants. These materials have very interesting inorganic–organic coordination structures that can be easily tailored to suit a specific application. Furthermore, their surface functionality can be improved by introducing other materials such as metal oxides, nanoparticles, and active functional groups, for the purpose of removing heavy metal ions. MOF composites that have been synthesized and modified for the adsorption of heavy metal ions have been characterized. Hence, in this review, we focussed on various analytical methods that have been employed in the reported studies of heavy metal adsorption. The combination of these techniques has provided a significant amount of data that assist in studying the behavior of MOF composites and their interactions with metal ions. Microscopic characterization techniques coupled with EDS/X were able to detect some elements that were adsorbed by MOFs. Further supports were also provided by the FTIR spectroscopy, wherein we observed the vibrational peaks of the introduced functional groups as well as a reduction in the peaks after adsorption. The XPS confirmed the adsorption of heavy metal ions by looking at the orbital of the active elements on MOF composites as well as the orbitals of the targeted metal ion. In conclusion, this review focussed on understanding the role that each analytical technique has in order to determine the efficiency of the adsorption process.

## Figures and Tables

**Table 1:** Different heavy metal detection techniques after the adsorption of heavy metal ions using MOF composites.

Prepared Adsorbent Material	Targeted Metal Ion Pollutant	Analytical Technique Used	Adsorption Capacity Determined from the Isotherm Model (mg g^−1^)	Ref.
Diaminomaleonitrile (DAMN)/MIL-101(Cr)	U(IV)	ICP-AES and ICP-MS	601	[58]
MIL-125-HQ	Pb(II), Cd(II), Cu(II), and Cr(III)	AAS	262.1, 102.8, 66.9, and 53.9	[59]
MIL-101(Cr)/Fe_3_O_4_@ADTC	Se(IV) and Se(VI)	Electrothermal (ET)-AAS	197	[60]
NH_2_-mSiO_2_@MIL-101(Cr) The	Cr(VI) and Pb(II)	UV-Vis and ICP-OES	73.2 and 161.3	[61]
MIL-101-PMIDA	Yttrium (Y) and lutetium (Lu)	ICP-MS	25.3 and 63.4	[62]
ED-MIL-101(Cr)	U(VI)	Trace uranium analyzer	200	[63]
ED-MIL-101(Cr)	Cu(II) and Cd(II)	Optical emission spectrometer	69.9 and 63.15	[64]
Ni_0_._6_Fe_2_._4_O_4_-UiO-66-PEI	Pb(II) and Cr(VI)	ICP-AES	273.2 and 428.6	[65]
SH@Cu-MOF	Hg(II)	FAAS	173	[66]

## Data Availability

Not applicable.
